# 

**Published:** 1889-12

**Authors:** 


					﻿astin  second clase matter
Vol. V
December 1889.
No. 6
DANIEL'S
■ ■	W^LJ^illV XL±?i
Journal
advince single
F.E. DANIEL
Von Stem
				

